# Bis{[1-(*tert*-butoxy­carbonyl)­pyrrolidin-2-yl]meth­yl} carbonate

**DOI:** 10.1107/S1600536809039452

**Published:** 2009-10-03

**Authors:** Lin-Hai Jing

**Affiliations:** a School of Chemistry and Chemical Engineering, China West Normal University, Nanchong 637002, People’s Republic of China

## Abstract

The asymmetric unit of the title compound, C_21_H_36_N_2_O_7_, consists of two independent half-mol­ecules, the other halves being generated by twofold rotational axes. The two independent half-mol­ecules are related by a pseudo-inversion center. In one, the pyrrolidine ring adopts a twist conformation whereas in the other it is in an envelope conformation. The crystal packing is stabilized by C—H⋯O hydrogen bonds.

## Related literature

For the use of proline derivatives in organocatalysis, see: Dalko & Moisan (2004 ▶). For the synthesis, see: Wiegrebe *et al.* (1974 ▶).
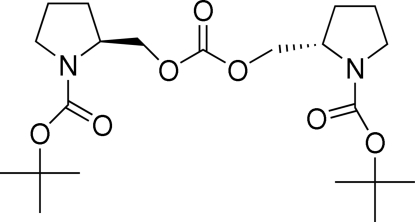


## Experimental

### 

#### Crystal data

C_21_H_36_N_2_O_7_
*M* *_r_* = 428.52
Monoclinic, 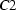

*a* = 21.995 (5) Å
*b* = 9.9534 (18) Å
*c* = 10.531 (2) Å
β = 100.631 (3)°
*V* = 2265.9 (8) Å^3^
*Z* = 4
Mo *K*α radiation
μ = 0.09 mm^−1^
*T* = 93 K
0.43 × 0.40 × 0.33 mm

#### Data collection

Rigaku AFC10/Saturn724+ diffractometer
Absorption correction: none
9271 measured reflections
2735 independent reflections
2473 reflections with *I* > 2σ(*I*)
*R*_int_ = 0.026

#### Refinement

*R*[*F* ^2^ > 2σ(*F* ^2^)] = 0.030
*wR*(*F* ^2^) = 0.073
*S* = 1.00
2735 reflections
280 parameters
1 restraint
H-atom parameters constrained
Δρ_max_ = 0.20 e Å^−3^
Δρ_min_ = −0.19 e Å^−3^

### 

Data collection: *RAPID-AUTO* (Rigaku, 2004 ▶); cell refinement: *RAPID-AUTO*; data reduction: *RAPID-AUTO*; program(s) used to solve structure: *SHELXS97* (Sheldrick, 2008 ▶); program(s) used to refine structure: *SHELXL97* (Sheldrick, 2008 ▶); molecular graphics: *XP* in *SHELXTL* (Sheldrick, 2008 ▶); software used to prepare material for publication: *SHELXL97*.

## Supplementary Material

Crystal structure: contains datablocks global, I. DOI: 10.1107/S1600536809039452/ci2927sup1.cif

Structure factors: contains datablocks I. DOI: 10.1107/S1600536809039452/ci2927Isup2.hkl

Additional supplementary materials:  crystallographic information; 3D view; checkCIF report

## Figures and Tables

**Table 1:** Hydrogen-bond geometry (Å, °)

*D*—H⋯*A*	*D*—H	H⋯*A*	*D*⋯*A*	*D*—H⋯*A*
C2—H2*B*⋯O2^i^	0.99	2.60	3.223 (2)	121
C5—H5*A*⋯O6^ii^	0.99	2.50	3.380 (2)	148
C13—H13*B*⋯O6^iii^	0.99	2.54	3.231 (2)	127

Symmetry codes: (i) 
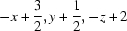
; (ii) 
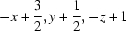
; (iii) 
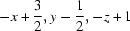
.

**Fig. 1.**
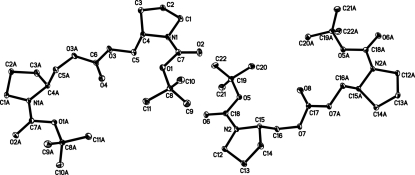
Views of the two independent molecules of the title compound, showing 30% probability displacement ellipsoids and the atomic numbering. Atoms labelled with the suffix A are generated by the symmetry operation (1-x, y, 1-z) in one of the molecules (with atom O1) and (2-x, y, 2-z) on the other (with atom O5).

## supplementary crystallographic information

### Comment

Proline derivatives are one of the most important catalysts in organocatalysis (Dalko *et al.*, 2004). Here, we report the crystal structure of the title compound.

The asymmetric unit of the title compound consists of one-half each of two crystallographically independent molecules (Fig. 1). The other halves are generated by crystallographic twofold rotational axes. Bond lengths and angles of these two molecules agree with each other and are normal. In one of the independent unit the pyrrolidine ring (N1/C1-C4) adopts a twist conformation whereas in the other the pyrrolidine ring (N2/C12-C15) adopts an envelope conformation.

The crystal packing is stabilized by C—H···O hydrogen bonds (Table 1).

### Experimental

The title compound was synthesized according to the method reported in the literature (Wiegrebe *et al.*, 1974). Colourless single crystals suitable for X-ray diffraction were obtained by slow evaporation of a methanol solution.

### Refinement

All H atoms were placed in calculated positions, with C-H = 0.98-1.00 Å, and refined using a riding model, with *U*_iso_(H) = 1.2*U*_eq_(C).

### Crystal data

**Table:**

C_21_H_36_N_2_O_7_	*F*(000) = 928
*M**_r_* = 428.52	*D*_x_ = 1.256 Mg m^−^^3^
Monoclinic, *C*2	Mo *K*α radiation, λ = 0.71073 Å
Hall symbol: C 2y	Cell parameters from 3700 reflections
*a* = 21.995 (5) Å	θ = 3.1–27.5°
*b* = 9.9534 (18) Å	µ = 0.09 mm^−^^1^
*c* = 10.531 (2) Å	*T* = 93 K
β = 100.631 (3)°	Block, colourless
*V* = 2265.9 (8) Å^3^	0.43 × 0.40 × 0.33 mm
*Z* = 4	

### Data collection

**Table:**

Rigaku AFC10/Saturn724+ diffractometer	2473 reflections with *I* > 2σ(*I*)
Radiation source: Rotating Anode	*R*_int_ = 0.026
graphite	θ_max_ = 27.5°, θ_min_ = 3.1°
Detector resolution: 28.5714 pixels mm^-1^	*h* = −28→28
multi–scan	*k* = −12→10
9271 measured reflections	*l* = −12→13
2735 independent reflections	

### Refinement

**Table:**

Refinement on *F*^2^	Secondary atom site location: difference Fourier map
Least-squares matrix: full	Hydrogen site location: inferred from neighbouring sites
*R*[*F*^2^ > 2σ(*F*^2^)] = 0.030	H-atom parameters constrained
*wR*(*F*^2^) = 0.073	*w* = 1/[σ^2^(*F*_o_^2^) + (0.041*P*)^2^ + 0.316*P*] where *P* = (*F*_o_^2^ + 2*F*_c_^2^)/3
*S* = 1.00	(Δ/σ)_max_ = 0.001
2735 reflections	Δρ_max_ = 0.20 e Å^−^^3^
280 parameters	Δρ_min_ = −0.19 e Å^−^^3^
1 restraint	Extinction correction: *SHELXL97* (Sheldrick, 2008), Fc^*^=kFc[1+0.001xFc^2^λ^3^/sin(2θ)]^-1/4^
Primary atom site location: structure-invariant direct methods	Extinction coefficient: 0.0009 (3)

### Special details

**Table:**

Geometry. All e.s.d.'s (except the e.s.d. in the dihedral angle between two l.s. planes) are estimated using the full covariance matrix. The cell e.s.d.'s are taken into account individually in the estimation of e.s.d.'s in distances, angles and torsion angles; correlations between e.s.d.'s in cell parameters are only used when they are defined by crystal symmetry. An approximate (isotropic) treatment of cell e.s.d.'s is used for estimating e.s.d.'s involving l.s. planes.
Refinement. Refinement of *F*^2^ against ALL reflections. The weighted *R*-factor *wR* and goodness of fit *S* are based on *F*^2^, conventional *R*-factors *R* are based on *F*, with *F* set to zero for negative *F*^2^. The threshold expression of *F*^2^ > σ(*F*^2^) is used only for calculating *R*-factors(gt) *etc*. and is not relevant to the choice of reflections for refinement. *R*-factors based on *F*^2^ are statistically about twice as large as those based on *F*, and *R*- factors based on ALL data will be even larger.

### Fractional atomic coordinates and isotropic or equivalent isotropic displacement parameters (Å^2^)

**Table:**

	*x*	*y*	*z*	*U*_iso_*/*U*_eq_	
O1	0.61523 (6)	0.82679 (13)	0.77609 (13)	0.0210 (3)	
O2	0.70399 (6)	0.83717 (14)	0.92900 (13)	0.0251 (3)	
O3	0.54724 (6)	1.15192 (13)	0.54879 (13)	0.0262 (3)	
O4	0.5000	0.9521 (2)	0.5000	0.0226 (4)	
N1	0.65682 (7)	1.02483 (17)	0.83522 (14)	0.0178 (4)	
C1	0.70038 (9)	1.1193 (2)	0.91204 (18)	0.0210 (4)	
H1A	0.7435	1.1000	0.9031	0.025*	
H1B	0.6973	1.1158	1.0046	0.025*	
C2	0.67903 (8)	1.2547 (2)	0.85289 (18)	0.0219 (4)	
H2A	0.6978	1.2741	0.7763	0.026*	
H2B	0.6894	1.3282	0.9165	0.026*	
C3	0.60872 (8)	1.23572 (19)	0.81495 (18)	0.0230 (4)	
H3A	0.5885	1.2452	0.8910	0.028*	
H3B	0.5905	1.3018	0.7484	0.028*	
C4	0.60199 (8)	1.09260 (18)	0.76148 (17)	0.0169 (4)	
H4	0.5634	1.0504	0.7804	0.020*	
C5	0.60269 (8)	1.0861 (2)	0.61761 (17)	0.0209 (4)	
H5A	0.6400	1.1319	0.5987	0.025*	
H5B	0.6036	0.9913	0.5896	0.025*	
C6	0.5000	1.0718 (3)	0.5000	0.0188 (6)	
C7	0.66228 (8)	0.8903 (2)	0.85346 (17)	0.0184 (4)	
C8	0.61266 (9)	0.6788 (2)	0.77050 (18)	0.0195 (4)	
C9	0.66997 (10)	0.6233 (2)	0.7267 (2)	0.0275 (5)	
H9A	0.7063	0.6376	0.7947	0.033*	
H9B	0.6759	0.6697	0.6478	0.033*	
H9C	0.6646	0.5269	0.7093	0.033*	
C10	0.60458 (10)	0.6224 (2)	0.90045 (19)	0.0248 (4)	
H10A	0.5703	0.6685	0.9296	0.030*	
H10B	0.6427	0.6361	0.9638	0.030*	
H10C	0.5956	0.5260	0.8918	0.030*	
C11	0.55506 (10)	0.6543 (2)	0.6687 (2)	0.0274 (5)	
H11A	0.5596	0.6988	0.5880	0.033*	
H11B	0.5188	0.6908	0.6988	0.033*	
H11C	0.5496	0.5576	0.6536	0.033*	
O5	0.87815 (6)	0.72148 (13)	0.73864 (12)	0.0195 (3)	
O6	0.79858 (6)	0.70687 (14)	0.56549 (12)	0.0224 (3)	
O7	0.97677 (6)	0.38716 (13)	0.90069 (11)	0.0193 (3)	
O8	1.0000	0.5876 (2)	1.0000	0.0284 (5)	
N2	0.84043 (7)	0.52083 (17)	0.67249 (14)	0.0172 (4)	
C12	0.79789 (8)	0.4284 (2)	0.59120 (17)	0.0198 (4)	
H12A	0.7987	0.4419	0.4984	0.024*	
H12B	0.7550	0.4393	0.6057	0.024*	
C13	0.82406 (10)	0.2922 (2)	0.63755 (19)	0.0240 (5)	
H13A	0.8583	0.2659	0.5935	0.029*	
H13B	0.7917	0.2219	0.6227	0.029*	
C14	0.84733 (9)	0.31487 (19)	0.78185 (18)	0.0235 (4)	
H14A	0.8789	0.2471	0.8173	0.028*	
H14B	0.8128	0.3104	0.8303	0.028*	
C15	0.87548 (7)	0.45583 (18)	0.78907 (16)	0.0156 (3)	
H15A	0.8680	0.5033	0.8684	0.019*	
C16	0.94416 (7)	0.45378 (19)	0.78534 (16)	0.0183 (4)	
H16A	0.9514	0.4052	0.7074	0.022*	
H16B	0.9597	0.5468	0.7815	0.022*	
C17	1.0000	0.4671 (3)	1.0000	0.0172 (5)	
C18	0.83557 (8)	0.6552 (2)	0.65213 (17)	0.0171 (4)	
C19	0.88191 (9)	0.8700 (2)	0.73624 (18)	0.0199 (4)	
C20	0.93749 (10)	0.8980 (2)	0.84211 (19)	0.0258 (4)	
H20A	0.9742	0.8549	0.8201	0.031*	
H20B	0.9298	0.8619	0.9243	0.031*	
H20C	0.9443	0.9951	0.8502	0.031*	
C21	0.89438 (10)	0.9192 (2)	0.60638 (18)	0.0270 (5)	
H21A	0.8577	0.9035	0.5395	0.032*	
H21B	0.9297	0.8702	0.5844	0.032*	
H21C	0.9037	1.0156	0.6117	0.032*	
C22	0.82306 (9)	0.9276 (2)	0.7694 (2)	0.0265 (4)	
H22A	0.8156	0.8876	0.8502	0.032*	
H22B	0.7882	0.9073	0.6997	0.032*	
H22C	0.8273	1.0252	0.7798	0.032*	

### Atomic displacement parameters (Å^2^)

**Table:**

	*U*^11^	*U*^22^	*U*^33^	*U*^12^	*U*^13^	*U*^23^
O1	0.0213 (7)	0.0133 (8)	0.0254 (7)	−0.0019 (5)	−0.0034 (5)	0.0023 (5)
O2	0.0217 (7)	0.0242 (9)	0.0260 (7)	0.0008 (6)	−0.0046 (6)	0.0067 (6)
O3	0.0272 (7)	0.0192 (7)	0.0259 (7)	−0.0017 (6)	−0.0118 (6)	0.0039 (6)
O4	0.0231 (9)	0.0178 (12)	0.0262 (10)	0.000	0.0024 (8)	0.000
N1	0.0147 (7)	0.0170 (9)	0.0193 (8)	−0.0025 (6)	−0.0027 (6)	0.0010 (6)
C1	0.0165 (9)	0.0244 (12)	0.0198 (9)	−0.0046 (8)	−0.0027 (7)	0.0003 (8)
C2	0.0190 (9)	0.0201 (11)	0.0242 (10)	−0.0026 (8)	−0.0021 (8)	−0.0018 (8)
C3	0.0189 (8)	0.0226 (10)	0.0259 (9)	0.0026 (8)	0.0000 (7)	−0.0038 (8)
C4	0.0120 (7)	0.0179 (10)	0.0201 (9)	−0.0004 (7)	0.0011 (6)	0.0010 (7)
C5	0.0169 (8)	0.0244 (10)	0.0197 (9)	0.0006 (7)	−0.0011 (7)	0.0013 (7)
C6	0.0212 (12)	0.0211 (16)	0.0131 (12)	0.000	0.0003 (10)	0.000
C7	0.0150 (8)	0.0222 (11)	0.0174 (8)	−0.0016 (8)	0.0011 (7)	0.0006 (8)
C8	0.0225 (9)	0.0136 (10)	0.0208 (10)	−0.0004 (8)	0.0002 (8)	0.0001 (7)
C9	0.0298 (10)	0.0281 (12)	0.0254 (10)	0.0028 (9)	0.0070 (8)	0.0013 (9)
C10	0.0323 (11)	0.0189 (11)	0.0241 (10)	−0.0016 (8)	0.0077 (8)	0.0016 (8)
C11	0.0307 (11)	0.0189 (11)	0.0286 (11)	−0.0009 (9)	−0.0053 (9)	−0.0011 (9)
O5	0.0218 (6)	0.0140 (8)	0.0201 (6)	−0.0001 (5)	−0.0032 (5)	−0.0006 (5)
O6	0.0211 (7)	0.0220 (8)	0.0215 (7)	0.0048 (6)	−0.0028 (6)	0.0028 (6)
O7	0.0184 (6)	0.0175 (7)	0.0187 (6)	0.0002 (5)	−0.0049 (5)	0.0004 (5)
O8	0.0403 (12)	0.0182 (12)	0.0235 (10)	0.000	−0.0028 (9)	0.000
N2	0.0160 (8)	0.0170 (9)	0.0160 (7)	0.0004 (6)	−0.0041 (6)	0.0005 (6)
C12	0.0184 (9)	0.0193 (11)	0.0192 (9)	−0.0021 (8)	−0.0031 (7)	−0.0018 (8)
C13	0.0250 (10)	0.0184 (12)	0.0244 (10)	−0.0052 (8)	−0.0062 (8)	−0.0005 (8)
C14	0.0230 (9)	0.0212 (10)	0.0236 (9)	−0.0057 (8)	−0.0029 (7)	0.0043 (8)
C15	0.0150 (7)	0.0159 (9)	0.0147 (8)	0.0012 (7)	−0.0003 (6)	0.0006 (7)
C16	0.0155 (8)	0.0223 (10)	0.0157 (8)	0.0007 (7)	−0.0008 (6)	0.0010 (7)
C17	0.0122 (11)	0.0201 (16)	0.0189 (12)	0.000	0.0017 (10)	0.000
C18	0.0165 (8)	0.0178 (11)	0.0169 (9)	0.0013 (7)	0.0030 (7)	0.0001 (7)
C19	0.0248 (10)	0.0127 (11)	0.0215 (9)	0.0014 (8)	0.0030 (8)	0.0015 (8)
C20	0.0296 (10)	0.0191 (11)	0.0268 (10)	−0.0037 (8)	−0.0003 (8)	−0.0023 (8)
C21	0.0345 (11)	0.0222 (12)	0.0246 (10)	−0.0019 (9)	0.0065 (9)	0.0017 (8)
C22	0.0286 (10)	0.0189 (11)	0.0331 (10)	0.0022 (8)	0.0086 (9)	−0.0040 (9)

### Geometric parameters (Å, °)

**Table:**

O1—C7	1.350 (2)	O5—C18	1.352 (2)
O1—C8	1.475 (2)	O5—C19	1.482 (2)
O2—C7	1.219 (2)	O6—C18	1.219 (2)
O3—C6	1.335 (2)	O7—C17	1.338 (2)
O3—C5	1.455 (2)	O7—C16	1.4519 (19)
O4—C6	1.191 (4)	O8—C17	1.200 (4)
N1—C7	1.355 (3)	N2—C18	1.355 (3)
N1—C4	1.472 (2)	N2—C12	1.469 (2)
N1—C1	1.473 (2)	N2—C15	1.473 (2)
C1—C2	1.522 (3)	C12—C13	1.518 (3)
C1—H1A	0.99	C12—H12A	0.99
C1—H1B	0.99	C12—H12B	0.99
C2—C3	1.536 (2)	C13—C14	1.528 (3)
C2—H2A	0.99	C13—H13A	0.99
C2—H2B	0.99	C13—H13B	0.99
C3—C4	1.529 (3)	C14—C15	1.530 (3)
C3—H3A	0.99	C14—H14A	0.99
C3—H3B	0.99	C14—H14B	0.99
C4—C5	1.519 (2)	C15—C16	1.518 (2)
C4—H4	1.00	C15—H15A	1.00
C5—H5A	0.99	C16—H16A	0.99
C5—H5B	0.99	C16—H16B	0.99
C6—O3^i^	1.335 (2)	C17—O7^ii^	1.338 (2)
C8—C10	1.520 (3)	C19—C22	1.514 (3)
C8—C11	1.521 (3)	C19—C20	1.520 (3)
C8—C9	1.524 (3)	C19—C21	1.524 (3)
C9—H9A	0.98	C20—H20A	0.98
C9—H9B	0.98	C20—H20B	0.98
C9—H9C	0.98	C20—H20C	0.98
C10—H10A	0.98	C21—H21A	0.98
C10—H10B	0.98	C21—H21B	0.98
C10—H10C	0.98	C21—H21C	0.98
C11—H11A	0.98	C22—H22A	0.98
C11—H11B	0.98	C22—H22B	0.98
C11—H11C	0.98	C22—H22C	0.98
			
C7—O1—C8	120.75 (16)	C18—O5—C19	120.63 (15)
C6—O3—C5	116.37 (15)	C17—O7—C16	116.16 (15)
C7—N1—C4	124.77 (16)	C18—N2—C12	120.16 (16)
C7—N1—C1	121.41 (17)	C18—N2—C15	125.37 (15)
C4—N1—C1	112.57 (16)	C12—N2—C15	112.93 (15)
N1—C1—C2	102.72 (15)	N2—C12—C13	102.06 (14)
N1—C1—H1A	111.2	N2—C12—H12A	111.4
C2—C1—H1A	111.2	C13—C12—H12A	111.4
N1—C1—H1B	111.2	N2—C12—H12B	111.4
C2—C1—H1B	111.2	C13—C12—H12B	111.4
H1A—C1—H1B	109.1	H12A—C12—H12B	109.2
C1—C2—C3	102.54 (16)	C12—C13—C14	103.02 (16)
C1—C2—H2A	111.3	C12—C13—H13A	111.2
C3—C2—H2A	111.3	C14—C13—H13A	111.2
C1—C2—H2B	111.3	C12—C13—H13B	111.2
C3—C2—H2B	111.3	C14—C13—H13B	111.2
H2A—C2—H2B	109.2	H13A—C13—H13B	109.1
C4—C3—C2	103.59 (15)	C13—C14—C15	104.08 (15)
C4—C3—H3A	111.0	C13—C14—H14A	110.9
C2—C3—H3A	111.0	C15—C14—H14A	110.9
C4—C3—H3B	111.0	C13—C14—H14B	110.9
C2—C3—H3B	111.0	C15—C14—H14B	110.9
H3A—C3—H3B	109.0	H14A—C14—H14B	109.0
N1—C4—C5	110.40 (14)	N2—C15—C16	110.76 (13)
N1—C4—C3	102.62 (14)	N2—C15—C14	102.50 (14)
C5—C4—C3	112.86 (15)	C16—C15—C14	112.45 (15)
N1—C4—H4	110.2	N2—C15—H15A	110.3
C5—C4—H4	110.2	C16—C15—H15A	110.3
C3—C4—H4	110.2	C14—C15—H15A	110.3
O3—C5—C4	108.41 (14)	O7—C16—C15	108.95 (13)
O3—C5—H5A	110.0	O7—C16—H16A	109.9
C4—C5—H5A	110.0	C15—C16—H16A	109.9
O3—C5—H5B	110.0	O7—C16—H16B	109.9
C4—C5—H5B	110.0	C15—C16—H16B	109.9
H5A—C5—H5B	108.4	H16A—C16—H16B	108.3
O4—C6—O3^i^	126.69 (12)	O8—C17—O7	126.48 (11)
O4—C6—O3	126.69 (12)	O8—C17—O7^ii^	126.48 (11)
O3^i^—C6—O3	106.6 (2)	O7—C17—O7^ii^	107.0 (2)
O2—C7—O1	126.3 (2)	O6—C18—O5	125.61 (19)
O2—C7—N1	123.91 (19)	O6—C18—N2	123.86 (19)
O1—C7—N1	109.83 (16)	O5—C18—N2	110.51 (16)
O1—C8—C10	110.09 (16)	O5—C19—C22	108.72 (16)
O1—C8—C11	102.07 (16)	O5—C19—C20	102.10 (15)
C10—C8—C11	110.79 (17)	C22—C19—C20	111.57 (16)
O1—C8—C9	110.27 (16)	O5—C19—C21	110.84 (16)
C10—C8—C9	112.29 (17)	C22—C19—C21	112.85 (17)
C11—C8—C9	110.88 (16)	C20—C19—C21	110.23 (17)
C8—C9—H9A	109.5	C19—C20—H20A	109.5
C8—C9—H9B	109.5	C19—C20—H20B	109.5
H9A—C9—H9B	109.5	H20A—C20—H20B	109.5
C8—C9—H9C	109.5	C19—C20—H20C	109.5
H9A—C9—H9C	109.5	H20A—C20—H20C	109.5
H9B—C9—H9C	109.5	H20B—C20—H20C	109.5
C8—C10—H10A	109.5	C19—C21—H21A	109.5
C8—C10—H10B	109.5	C19—C21—H21B	109.5
H10A—C10—H10B	109.5	H21A—C21—H21B	109.5
C8—C10—H10C	109.5	C19—C21—H21C	109.5
H10A—C10—H10C	109.5	H21A—C21—H21C	109.5
H10B—C10—H10C	109.5	H21B—C21—H21C	109.5
C8—C11—H11A	109.5	C19—C22—H22A	109.5
C8—C11—H11B	109.5	C19—C22—H22B	109.5
H11A—C11—H11B	109.5	H22A—C22—H22B	109.5
C8—C11—H11C	109.5	C19—C22—H22C	109.5
H11A—C11—H11C	109.5	H22A—C22—H22C	109.5
H11B—C11—H11C	109.5	H22B—C22—H22C	109.5
			
C7—N1—C1—C2	176.45 (17)	C18—N2—C12—C13	174.22 (16)
C4—N1—C1—C2	−15.76 (19)	C15—N2—C12—C13	−19.19 (18)
N1—C1—C2—C3	33.84 (18)	N2—C12—C13—C14	35.11 (18)
C1—C2—C3—C4	−40.29 (18)	C12—C13—C14—C15	−39.24 (19)
C7—N1—C4—C5	−81.3 (2)	C18—N2—C15—C16	−78.9 (2)
C1—N1—C4—C5	111.38 (17)	C12—N2—C15—C16	115.30 (16)
C7—N1—C4—C3	158.15 (17)	C18—N2—C15—C14	160.90 (17)
C1—N1—C4—C3	−9.15 (19)	C12—N2—C15—C14	−4.86 (18)
C2—C3—C4—N1	30.20 (18)	C13—C14—C15—N2	26.95 (18)
C2—C3—C4—C5	−88.61 (17)	C13—C14—C15—C16	−92.03 (17)
C6—O3—C5—C4	−98.00 (16)	C17—O7—C16—C15	−94.52 (15)
N1—C4—C5—O3	178.94 (14)	N2—C15—C16—O7	−179.11 (14)
C3—C4—C5—O3	−66.87 (18)	C14—C15—C16—O7	−65.08 (18)
C5—O3—C6—O4	−5.10 (16)	C16—O7—C17—O8	−5.21 (14)
C5—O3—C6—O3^i^	174.90 (16)	C16—O7—C17—O7^ii^	174.79 (14)
C8—O1—C7—O2	−4.2 (3)	C19—O5—C18—O6	1.8 (3)
C8—O1—C7—N1	175.71 (15)	C19—O5—C18—N2	−179.82 (14)
C4—N1—C7—O2	−169.34 (17)	C12—N2—C18—O6	−2.7 (3)
C1—N1—C7—O2	−3.1 (3)	C15—N2—C18—O6	−167.50 (16)
C4—N1—C7—O1	10.8 (3)	C12—N2—C18—O5	178.84 (14)
C1—N1—C7—O1	176.99 (15)	C15—N2—C18—O5	14.0 (2)
C7—O1—C8—C10	64.9 (2)	C18—O5—C19—C22	65.4 (2)
C7—O1—C8—C11	−177.36 (15)	C18—O5—C19—C20	−176.56 (15)
C7—O1—C8—C9	−59.5 (2)	C18—O5—C19—C21	−59.2 (2)

Symmetry codes: (i) −*x*+1, *y*, −*z*+1; (ii) −*x*+2, *y*, −*z*+2.

### Hydrogen-bond geometry (Å, °)

**Table:**

*D*—H···*A*	*D*—H	H···*A*	*D*···*A*	*D*—H···*A*
C2—H2B···O2^iii^	0.99	2.60	3.223 (2)	121
C5—H5A···O6^iv^	0.99	2.50	3.380 (2)	148
C13—H13B···O6^v^	0.99	2.54	3.231 (2)	127

Symmetry codes: (iii) −*x*+3/2, *y*+1/2, −*z*+2; (iv) −*x*+3/2, *y*+1/2, −*z*+1; (v) −*x*+3/2, *y*−1/2, −*z*+1.
